# Last Mile Access to Enriched Children's Complementary Food: Mitigating Malnutrition in Kenya

**DOI:** 10.3389/fpubh.2021.604864

**Published:** 2021-02-25

**Authors:** Muthuuri Pamela, Kassim Joseph, Richard Patrick, Mwarania Florence

**Affiliations:** ^1^Global Integrated Innovations Africa, Nairobi, Kenya; ^2^Faculty of Law, Economics and Governance, Utrecht School of Economics, Utrecht University, Utrecht, Netherlands; ^3^School of Public Health, Uniformed Services University of the Health Sciences, Bethesda, MD, United States

**Keywords:** child nutrition, last mile, complementary food, social entrepreneurship, child development

## Abstract

Commercial complementary foods are not accessible at the last mile of delivery, despite a veritable stunted growth explosion in Kenya. A Mile for the Brain aims to reduce child malnutrition by solving the pervasive distribution bottlenecks and prohibitive pricing challenges. This paper presents the systematized measurement for change process. We focus on the selection of off-the-shelf complementary foods, training of women entrepreneurs responsible for commercializing these complementary foods, coaching given to mothers on appropriate feeding education, and lastly the learning cycle revolving around feeding mothers, entrepreneurs for the Mile for the Brain social enterprise itself. We highlight the real-life challenges involved in this process in the context of adversity and constrained resources. The results, findings, and policy implications of this study will be reported elsewhere.

## Introduction

Stunting affects over 165 million children globally (1). It is an important contributor to the population-attributable risk of child mortality, overall burden of disease, and failure to reach full developmental potential or sub-optimal development in children characterized by delayed cognitive, motor and socio-emotional development (2, 3). Several approaches to combat stunting have been attempted, ranging from exclusive assistance programs such as refugees, people affected by man-made or disaster-related emergencies; to populations requiring food assistance during lean or poor harvest periods; to populations who are not typically food insecure but consume a relatively monotonous diet. Nutritious foods, including ready-to-use complementary foods (RUCFs) have been shown to increase child survival in chronic malnourished children (4).

This study's primary objective is to improve early childhood development through a complementary feeding program that prevents malnutrition in children aged 6–24 months. We hypothesize that increasing access to complementary foods will improve early childhood development including cognitive, motor, and sensory functions. Our unique business model attends to the availability and demand for complementary foods through repackaged affordable sizes in poor communities in Meru and Bungoma counties in Kenya from 2014 to 2016. The purpose was to empower rural women economically as a secondary objective.

## Background

Approximately 200 million children under 5 years of age in developing countries are not reaching their full development potential (1). Studies have categorized childhood development risk factors as being *biological* (such as nutrition deficiency, which leads to stunting, inadequate cognitive stimulation, iron deficiency and iodine deficiency), *environmental* (for example exposure to arsenic or lead), *psychosocial* (maternal depression, exposure to violence and parenting factors), and *infections* (including HIV infections that lead to severe hydrocephalus) (5). When exposed to any of these risk factors, children younger than 2 years of age are severely affected, as vital development—especially of the brain which controls cognitive, motor, and sensory functions of the human body—is developing rapidly during this period (5). Any small disturbance in processes occurring at this time can have long-term effects on the brain's structural and functional capacity (5). Ultimately, these effects amount to wasted human capital and hinder national development, translating to a cycle of poverty across generations (6).

The World Health Organization (WHO) defines an infant as stunted, wasted, or underweight if their Z-score for -length-for-age; weight-for-length; or weight-for-age, respectively, is less than a standard deviation of (–)2 (7). The main causes of growth stunting are chronic malnutrition, chronic or recurrent infections (with or without intestinal parasites), low birth weight, and extreme psychosocial stress without nutritional deficiencies (8). Stunted children have a likelihood of frequent episodes of severe diarrhea and are more susceptible to several infectious diseases, such as malaria, meningitis, and pneumonia, which result in increased fatality risk (9).

Among preventive measures that would reduce stunting in children under the age of 5 years, exclusive breast-feeding and good quality complementary feeding are ranked first and third, respectively (10). Studies have shown that poor-quality complementary foods and inappropriate feeding practices are among the major causes of malnutrition in young children (11, 12). In many developing countries, complementary foods are introduced too early or too late, with insufficient quality and quantity, leading to a great risk of nutritional deficiencies during the second half of infancy.

In developing countries like Kenya, home preparation of complementary weaning foods containing proteins has not been widely adopted possibly due to its higher cost and longer time required to prepare (13). Presently, in the attempt to lower costs of complementary foods there is a notable shift to producing complementary feeds using blends of cereals and plant-based protein-rich legumes like soybeans and cowpeas (14). Legume blend foods are more nutritious complementary foods however, they cost more than plain cereal flours like corn because of the higher cost of production (15). Nutritious, low cost, commercially packaged foods are a more viable alternative to homegrown complementary feeds. These specifically formulated foods include fortified blended foods where key vitamins and minerals are added to cereals, commercial infant cereals, and ready-to-use foods such as pastes, compressed bars and biscuits (16).

Ready to use complimentary foods are extremely convenient in preventing stunting (17). Consumption, storage and preparation is least involving to the users (17). Moreover, complimentary foods are easy to store and transport over long distances because they contain very little or no water which prevents mold and bacteria growth. Women in multiple developing countries have time and economic constraints with numerous agricultural and other competing domestic responsibilities, in addition to feeding and caring for their children (18). Saving time on food preparation could ensure that children 6–24 months old are fed frequently each day, thus contributing to the reduction in child under-nutrition. Where availability of cooking fuel or time for food preparation is limited, providing ready-to-use food for individuals suffering from stunting is more effective compared to providing a meal that needs preparation in addition to cooking the family's main meal (19, 20).

However, commercially distributed ready to use complimentary foods are still not reaching the most vulnerable population segments, due to high prices or the limited extension of commercial distribution systems into rural areas. Distribution bottlenecks or what is sometimes referred to as the “tragedy of the last mile” continues to be a major hindrance to access of essential supplies in many remote areas in sub-Saharan Africa (21). This is a consequence of poor road infrastructure, poor supply chains, lack of economies of scale and presence of multiple middlemen (intermediaries), thus, increasing the cost of essential products to the poor. Insufficient information feedback to the source of the product further results in weak supply-chain efficiencies (22). The distribution of bulky essential commodities like complementary foods for infants is even a bigger challenge.

A “Mile for the Brain” project aims to use an innovative business model to increase the use of ready to use complimentary foods and other nutritious foods in remote and poor areas of Kenya from 2014 to 2016. We will then assess the project impact on child growth and development. The overarching outcome of this project is to ascertain whether packaged complementary foods reduce mild or moderate malnutrition in the vulnerable 6–24 months old children in poor rural areas of Kenya. In this paper, we examine the real-life challenges experienced when carrying out the intervention in the context of adversity and constrained resources. We also report the changes we made along the way in optimizing our measurement efficacy.

## Description of the Intervention

The study was be a cluster-randomized controlled trial featuring an active control group within a sample size of 269 children and 300 women social entrepreneurs. The project was carried out in Meru and Bungoma Counties of Eastern and Western Kenya, respectively, and leveraged habits developed in these communities in 2014–2016. Clusters were selected based on three levels. First, 2 constituencies were randomly selected in each county. Secondly, 4 wards in each constituency were randomly selected. Lastly, 10 villages were randomly selected within the wards. The sample was identified by going to every other house in the village, after randomly determining the starting direction by the flip of a coin. In observance of the intent-to-treat approach, the treatment and control groups were not selected a priori, but were selected depending on the uptake of the intervention. Dyads were assigned to the intervention group if they consistently bought three or more sachets of food for a cumulative period of 3 months, while the control group are those who did not purchase any or purchased less than three sachets of food for a cumulative period of 3 months. The control group utilized traditional weaning complementary foods that are known to lack adequate micro-nutrient density such as corn. There were no socio-demographic differences observed between the treatment and control group at baseline; hence, minimizing any potential biases in the outcomes

The project engaged women to reach out to other women. They were provided with business and nutritional training skills. The women were engaged in door-to-door marketing of the re-packaged product to mothers with infants while educating them on the benefits of weaning infants on ready to use complementary foods. Some of the women in the groups were mothers of infants and served as good resource in informing on how the project was rolling out. The project leveraged these women's network groups to market and distribute the complementary foods.

In poor parts of Meru and Bungoma, similar to other rural communities in Kenya, there is a “kadogo” (small unit) economy that thrives on mass marketing using small sized packaging. Buyers purchase essential commodities in quantities at a price that they can afford. Margarine, detergents, soaps, etc. are halved and quartered depending on the customer's economic ability. Big consumer goods companies have repackaged their products in order to profit from this mass market. This approach is suited for fast moving, low value, high frequency products and has not been tried for delivery of slow-moving bulky products like complementary foods. Making ready to use complimentary foods available and accessible through this “kadogo economy” is anticipated to give poor households the option for proper complementary feeding of their children beyond cooking limited starch dense foods. The objective is to do so in a sustainable way, with revenues from product sales being enough to cover variable costs. Such ready to use complimentary foods will increase nutrient intake and save caretakers time taken for preparation in both rural and urban areas. Children will be fed nutritious foods at least three times each day.

Complementary foods were made available to the treatment group of 269 caregiver-baby dyads. The control group utilized traditional weaning complementary foods that are known to lack adequate micro-nutrient density such as corn. Changes in measures of physical development, anthropometric measurements, neuro-cognitive development, gross/fine motor skills, cognitive function, receptive language, expressive language, and socio-emotional capacities in children aged 0–24 months were assessed to determine the long-term well-being of the child. Anthropometric measurements were taken during each visit and included naked weight, recumbent length, and head circumference were measured. A culturally informed measure of developmental outcome, KDI—Kilifi Developmental Inventory Tool (23). The KDI consists of 69 items, calculated as a score for locomotor skills and eye–hand co-ordination. A second tool, the Developmental Milestones checklist (DMC_II) was used to provide motor, language and personal-social scores for children aged from 3 to 24 months.

## Project Design and Implementation

### Theory of Change

If mothers and/or caregivers of children in rural areas were educated on age-appropriate feeding and had access to high protein and fortified complementary food for purchase, they would then have a predilection for these foods for their children, reducing the risk for child malnutrition (24).

**Figure 1:**
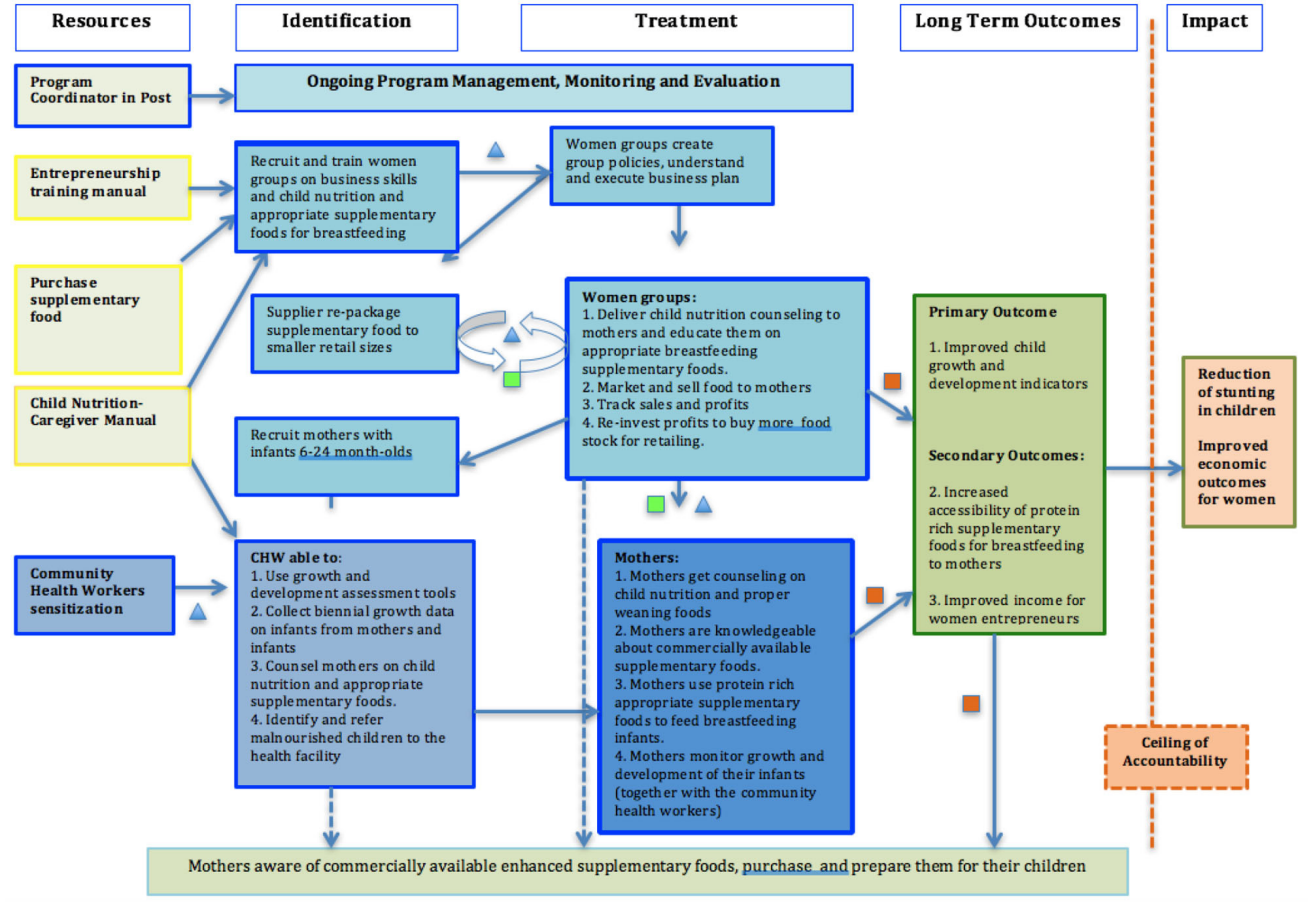
Theory of change.

When context-specific infant feeding messages promoting the use of local foods are delivered directly to mothers through counseling, significant improvements in complementary feeding practices and dietary intake are possible (24). The following theory of change (Figure 1) framework guided the design and the implementation of the project.

In this theory of change, women entrepreneurs were trained on business skills, to encourage entrepreneurship and growth of business, good bookkeeping. The women entrepreneurs were also trained on appropriate child nutrition, knowledge they used for their sales pitch of the baby complementary food, and to educate potential buyers. The women entrepreneurs were then provided with three choices of baby porridge flour to sell within their social networks. The sale of baby flour was done as a supplementary income activity, as the women entrepreneurs carried out their routine daily activities, and encountered mothers of babies. Mothers of babies benefited through age appropriate nutrition education they received from women entrepreneurs, and also through convenient access of the baby flour delivered to them by the women entrepreneurs for purchase. The primary outcome was to improve child growth and development outcomes.

### Study Objectives

Our objective was to capture measurement for change at the design and planning level. We sought out to identify assumptions embedded within the theory. In particular, the barriers to mothers/caregivers in accessing high protein enhanced complementary food for their children. Additionally, we studied the interactions between food suppliers, women entrepreneurs, and mothers/caregivers to better understand the source of variability in outcome. We identified and reported unanticipated game changers in the course of the project be identified to ensure support of the targeted groups (mothers/caregivers, women entrepreneurs) so as to achieve the intended outcome of reduced child malnutrition.

## Methods

### MEL Framework and Design

Approach for the monitoring and learning framework was to ensure adequate information is available for activity management and that data collection is consistent with data and learning needs of the Project. It describes the process for monitoring, evaluating, and learning from implementation to adapt and achieve results, and to know whether an activity is making progress toward stated results.

In collaboration with partners (Ministry of Health, Ministry of Agriculture, Healthcare facilities) and representatives target communities (women entrepreneurs and mothers/caregivers), we drafted the main MEL activities. The project team then put together a plan for undertaking the MEL activities. The activities of the MEL plan were guided by the project theory of change, Figure 1: Theory of Change.

### Data Collection and Management

Intervention activities, selling and purchase of complementary food for babies 6–24 months were initiated in Meru County, Kenya. When making the sales pitch, the women sellers educated the mothers buying the food on age-appropriate feeding and the benefits of weaning infants on ready to use complementary foods. Post data collection, participants were assigned in the intervention group if they consistently bought three or more sachets of food for a cumulative period of 3 months, while the control group are those who utilized traditional weaning complementary foods that are known to lack adequate micro-nutrient density such as corn. Data was collected from the four domains of operations, women entrepreneurs, mothers/caregivers, and babies through survey data forms, and also unstructured qualitative feedback. Data forms were completed using an electronic device, a computer tablet. Data was entered directly in this electronic device during service delivery. A field supervisor verified the data integrity weekly applying necessary measures to ensure clean data. The data was stored in a stable and secure server, and backup server data on a daily basis. The project was approved by Moi University Faculty of Health Sciences Institutional Review Board (IRB).

Data for learning was generated through (1) impact surveys collected at baseline and end-line data, and through (2) routine monitoring and evaluation, process assessment collected weekly, bi-weekly or monthly. Monitoring and evaluation data created multiple iterations of feedback and learning about the delivery of the intervention activities, which triggered changes throughout the design and implementation phases of the project.

**Figure 2:**
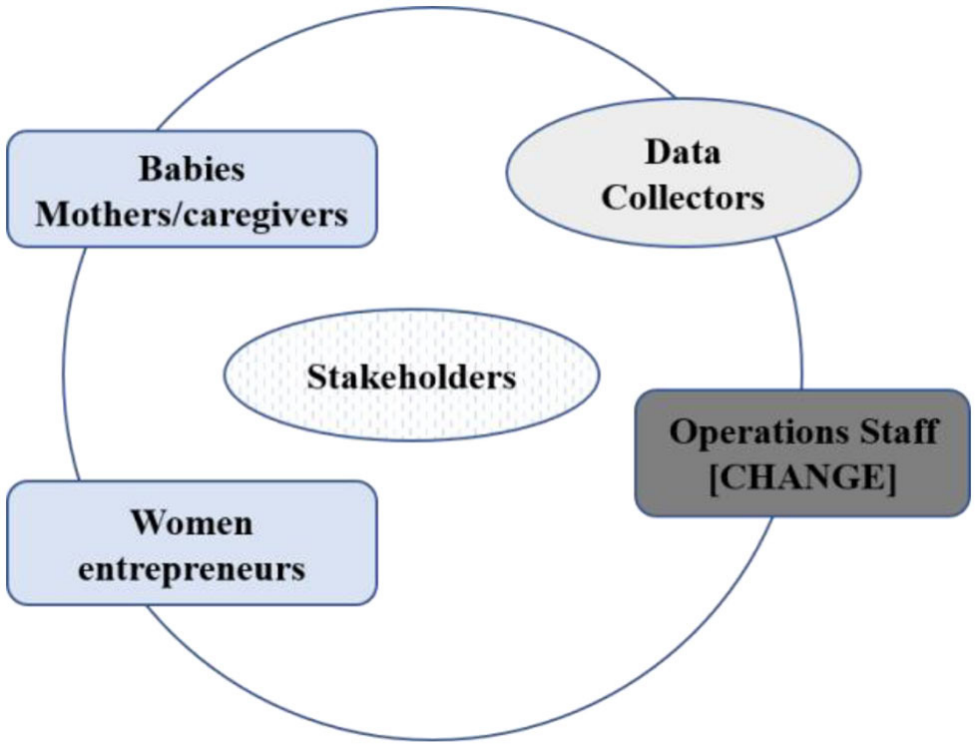
Schematic showing the structure of the learning and feedback loop. The MEL plan has 4 levels that are interconnected; Level 1: Operations Level, 2: Women Entrepreneurs, Level 3: Babies Caregiver/Mother, and Level 4: Data Collectors.

In general, the flow of data or information was a feedback cyclic loop with a two-way data origination (Figure 2). The main flow of data was from Babies, Mothers/Caregivers, being our target beneficiaries, to women entrepreneurs, who would then submit the data reports to the operations staff on a routine basis or to data collectors periodically during scheduled data collection engagements. This data was structured, based on established indicators, or unstructured based on real time experience with the project. Women entrepreneurs also generated their own data based on their specific engagement of the project, the marketing of the baby porridge flour. Periodically, data staff would collect scheduled data at certain points during the implementation phase, such as baseline data, quarterly and end line.

Stakeholders (collaborating partners such as the Ministry of Health, Ministry of Agriculture, Kenya Women's Finance Trust) and beneficiaries (babies, mothers/caregivers, women entrepreneurs) were involved in making the changes to ensure relevance and responsiveness in the administrative processes, and in the implementation of intervention activities.

There was a total of 262 caregiver/baby dyads from an original 269 dyads, there was an attrition of 7 dyads, resulting in negligible impact on the results. Data or information from caregivers was sought directly from them by data collectors or by women entrepreneurs, who would then rely the information to the data collectors and operations staff. There were 300 women entrepreneurs organized in economic units of 10 members per group. Representatives of the group (chairperson, treasurer, secretary), were frequently the sources of information on behalf of the group.

## Discussion

Assessments to establish a causal link between complementary feeding interventions and developmental outcomes are difficult to evaluate as there are numerous methodological problems. Complementary feeding practices encompass a series of inter-related behaviors that must be considered simultaneously and are therefore difficult to summarize into one or a few variables that accurately reflect these practices (25).

Optimal complementary feeding practices depend not only on what is fed, but also on how, when, where, and by whom the child is fed by. Thus, baseline assessments should be conducted to identify gaps in knowledge and current good practices. They should be followed by trials of education message options for improving the diet and feeding practices, identification of priority audiences, and effective strategies for reaching these audiences.

This discussion is a reflection on our measurement for change process, what we learnt, and how our processes helped the kinds of reviews and changes in the different approaches we used in designing and implementing our study to achieve maximum impact.

In the project design stage, we created a theory of change, to improve access to high nutrients complementary food, the main approaches were to create awareness of the availability of commercially ready to eat complementary food, a concept that is not common in the last mile. Once this awareness is created by women entrepreneurs through their nutrition education and sales pitch, these women give mothers the option of three highly nutritious readily available foods already retailing in the market that they can purchase at affordable prices. In the design, the assumption was to provide choice to the mothers, however the mothers on their own singled out one product. The women brought the food to the mothers for purchase.

In the learning cycle, through the women entrepreneurs, we received feedback that one of the three selected foods was rejected by the mothers because their children did not like the taste, and also made them uncomfortable and cry. The second food was also rejected by the mothers because the price was slightly higher. That left us with the food that was selected by the mothers as optimum for the price and acceptability by the babies.

Envisioned in the original theory of change, women entrepreneurs were to repackage bulky complementary foods into small affordable sachets for sale within their networks. In examining the context, together with local stakeholders in the Ministry of Health and other nutrition experts, we identified the challenge to maintain hygiene, and require close supervision if repackaging of food was done by the women, a challenge that can easily become an obstacle during scale up. Instead, we negotiated with the food manufacturer to do the packaging into small sachets at the plant. This change ensures adequate food hygiene is adhered to, and also eliminates the need for additional labor to repackage and supervise once the food reached the women entrepreneurs.

Women entrepreneurs were recruited in functioning economic units of 10 members per group. These were already existing active groups, with the assumption that they had already overcome group dynamics therefore effective and efficient in carrying out the group activities of selling food. Therefore, all group members had to sell all their assigned food units but pooled their money together to replenish their stock or expand their stock capital. This assumption was challenged, when we received feedback from women entrepreneurs during monthly meetings with the group officials (chairperson, secretary, treasurer), that some members of a given group outperformed the others and sold all their food within days, while others took a week or two. Based on this feedback, we dissociated the requirement for a group to function as an economic unit except for administrative purposes. The change enabled individuals to function as economic units and did not limit their individual economic potential by functioning as a group.

A Mile for the Brain nutrition campaign targeted children aged 6–24 months, specifically improving their consumption of high protein in their complementary feeding. The assumption was that mothers would only feed their children who are within this age group. However, mothers prepared the food for all their children including the older ones (5 years and above). Since it was the mother's choice, we modified the campaign to emphasize the need for mothers to feed the right quantities to children 6–24 months, and then feed the older children of what remains.

A mile for the brain project was envisioned as a woman driven change, with mothers and female caregivers as the benefactors. The approach leveraged women entrepreneurs' social networks with other women/caregivers during their day-to-day interactions such as in worship gatherings, on the road, neighborhood, friends and relatives. Once the project had a stronghold, men in the community got interested and wanted to be involved in the sale of complementary foods. We revised the approach to incorporate the male model to ensure men are represented as caregivers and head of household.

Although women were the primary caregivers and the sole preparer of food for the family, it was also noted in some households that fathers had the ultimate say on whether the baby should be fed the enhanced complementary food. Some fathers were opposed to the food and wanted their children to only be fed the traditional foods. The changes created by this feedback cycle resulted in fathers being involved in sales pitches. They were also educated about the benefits of the age-appropriate use of enhanced complementary food for babies 6–24 months. This nutrition education for fathers enriched their depth in feeding decisions.

Women entrepreneurs educated mothers on how to prepare food for their babies, and also assisted some of them through demonstrations. However, some mothers would use more water than required to stretch multiple feedings. This situation compromised or dilutes the nutrients expected to be consumed by the baby per meal. This prompted the project to incorporate random audit visits by community health workers to observe these mothers while they prepare the complementary foods and re-educate those who were doing it incorrectly.

The intervention showed greater improvement in growth outcomes for participating children compared to non-participants: mothers of children with lower age adjusted Mid Upper Arm Circumference z-scores were 62.5% more likely to purchase food than those with higher z-scores [odds ratio 1.625 (0.53–4.98) at 95% confidence interval]. Children of mothers who purchased enhanced complimentary food performed better than those that did not, in the age adjusted means of eye-hand coordination and locomotor scores for the KDI playing with the ball test (*p* < 0.10). Age adjusted means scores for the personal/social test using DMC-II were higher among children of those who purchased food than those who did not purchase (0.121 vs. −0.134) at 95% C.I.

## Conclusion

Measurement for change is critical in all stages of implementation. It is therefore important to build a culture of measurement for change from the very beginning, at the design phase of the project. With proper planning, measurement for change creates efficiency in use of the time and resources in the long run, however, it does require thoughtful build-in for time and other resources to enable it to be performed successfully.

## Data Availability Statement

The original contributions presented in the study are included in the article/supplementary material, further inquiries can be directed to the corresponding author/s.

## Ethics Statement

The studies involving human participants were reviewed and approved by Moi University, Faculty of Health Science, Kenya. Written informed consent to participate in this study was provided by the participants' legal guardian/next of kin.

## Author Contributions

All authors listed have made a substantial, direct and intellectual contribution to the work, and approved it for publication.

## Conflict of Interest

The authors declare that the research was conducted in the absence of any commercial or financial relationships that could be construed as a potential conflict of interest.
